# Effect of a short video on patients’ motivation for dose reduction or cessation of hypnotics

**DOI:** 10.1007/s41105-023-00446-4

**Published:** 2023-01-27

**Authors:** Misato Amagai, Motohiro Ozone, Tomohiro Utsumi, Ayana Hotchi, Masayuki Iwashita, Wataru Yamadera, Masahiro Shigeta

**Affiliations:** 1 Department of Psychiatry, The Jikei University School of Medicine, Tokyo, Japan; 2 Department of Neuropsychiatry, Kurume University School of Medicine, 67 Asahi-Machi, Kurume City, Fukuoka Prefecture 830-0011 Japan

**Keywords:** Dose reduction, Educational video, Hypnotics, Insomnia, Motivation, Sleep hygiene

## Abstract

Recently, addiction to regular doses of hypnotics has become a problem. While many patients report a desire to reduce their dosage, many do not agree with their physicians’ suggestions for reduction. In this study, we created an online short video targeting patient motivation to reduce hypnotics usage and examined its efficacy and factors associated with the intention to reduce medication. We created a 10 min video that included “sleep education,” “guidance for reducing the use of hypnotics,” and “systematic motivation for reducing the use of hypnotics” and posted it on our website. For 1 year, we conducted a questionnaire survey to determine the effectiveness of the video and factors associated with the intention to reduce hypnotics use. Of 4548 viewers, 609 (13.4%) completed the questionnaire, 369 (67.9%) of whom used hypnotics. Most respondents were older adults. The intention to reduce medication use was significantly strengthened after watching the video in 37.7% of medication users (effect size 0.404). In the group that was not inclined toward medication reduction before viewing, 85.2% of patients had stronger intentions to reduce medication use after watching the video (effect size 0.818). “Memorable content about side effects” was extracted as a factor related to reinforcement of the intention to reduce medication use, suggesting that prescribing physicians’ descriptions of current insomnia treatment is inadequate in explaining side effects to patients. A short informational video can have beneficial effects on patients’ motivation for dose reduction or cessation of hypnotics.

**Supplementary Information:**

The online version contains supplementary material available at 10.1007/s41105-023-00446-4.

## Introduction

Insomnia is a common disease and medication is a popular treatment, as it provides rapid relief. Although hypnotics are practical for acute insomnia, they can have serious side effects, including falls, carryover effects, and memory impairment [1]. International guidelines recommend short-term, minimal use [2].

Insomnia has a complex pathophysiology, including genetic, environmental, behavioral, and physiological factors [3], and its occurrence increases with age [4]. It is generally assumed that the causes of insomnia are different in cognitive mechanism between younger and older patients. While younger people often have sleep disorders due to poor sleep hygiene and circadian rhythm problems (delayed sleep phase) [5], elderly people are often medicated due to the mistaken belief that insomnia is normal aging phenomenon [6]. Regular sleep medication is linked to dependence via gamma-aminobutyric acid receptor agonists [7, 8], making users psychologically resistant to dose reduction. Conversely, in a study involving 600 people in the United Kingdom of intentions to reduce hypnotic use, one in five people wanted to stop using hypnotics, and one in two had attempted cessation in the past [9]. This suggests that users of hypotics are ambivalent, that is, both willing and unwilling to reduce their medication.

Insomnia is often treated by a general practitioner [10]. A survey of the current state of primary care in the United Kingdom reported cases of sudden hypnotic discontinuation without adequate withdrawal interventions [11]. Inappropriate hypnotic use, without withdrawal strategies, can lead to withdrawal symptoms and dependence [12]. Many patients who once experienced withdrawal symptoms and decreased self-efficacy upon hypnotic discontinuation became nervous, hypervigilant, reluctant to reduce their medication, and required special consideration [13].

In many clinical cases, suggestions to reduce the medication dose by physicians are rejected. In such cases, motivating patients to reduce their medication is an effective strategy. Common sleep hygiene guidance alone is not successful in treating insomnia [14], although distribution of educational booklets can reduce long-term benzodiazepine use [15]. A letter or single general practitioner consultation for long-term users can assist in reducing or stopping medication [16]. This suggests that education, even if it is a unidirectional one-time occurrence, can increase willingness to reduce medication.

This study examined the effectiveness of a short video targeting motivation for sleep medication withdrawal, which was posted on the internet. Furthermore, we aimed to contribute to the proper use of hypnotics by examining associated factors in patients who improved their intention to reduce their medication use, or not.

## Materials and methods

### Subjects

We surveyed the public respondents who watched the video, and analyzed the data from those who voluntarily answered the questionnaire. The video was published by the Department of Psychiatry, Jikei University School of Medicine (https://jikei-psy.com/zzz). The survey period lasted from February 1, 2019, to January 31, 2020.

### Video creation

To motivate people to reduce their hypnotics use, we created a 10 min, 11 s video containing the following: (1) sleep education, basic knowledge of sleep, and sleep hygiene instructions; (2) dose reduction guidance, specific methods, and precautions for dose reduction; and (3) motivation for dose reduction and encouragement to change intentions, with reference to the behavior change model [17]. We explained the content in plain language using animation, empathizing with the viewers’ conflicts and distress, and introducing supportive comments using a psychiatric approach.

### Measurements

An online survey was included on the same web page as the video. Viewers were asked to complete the questionnaire after viewing the video (Online Resource 1; Questionnaire form).

### Items

Items listed in the questionnaire included: age, sex, medication status, insomnia symptoms, reason for viewing, comprehension, intention to reduce their medications before and after viewing the video, intention to consult their doctor, and memorable content of the video (Table 1).

**Table 1:** Items in the questionnaire and their options

	Items	Options
1. Fundamental information	Age	Teens/20 s/30 s/40 s/50 s/60 s/70 s/Over 80
	Sex	Female/Male
	Medication Status	Non/Daily/Only necessary
2. Insomnia symptoms	Difficulty in falling asleep	None /Mild/Moderate/Severe/Very severe
	Mid-arousal	None/Mild/Moderate/Severe/Very Severe
	Early morning awakening	None/Mild/Moderate/Severe/Very severe
	Daytime dysfunction (e.g., daytime fatigue, ability to perform work daily chores, concentration, memory, mood, etc.)	None/A little/Somewhat/Much/Very much
3. Video viewing	Reason for viewing the video	Recommendation from my doctor/Recommendation from acquaintances/By chance/Have trouble sleeping/Interested in sleep medicine/Have anxiety about taking sleep medication/Others
	Comprehension of the video	Fully understood/Understood/Partially did not understand/Hardly understand/Did not understand at all
	Intention to reduce the dose before viewing the video	Strongly agree/Agree/Neither agree nor disagree/Disagree/Strongly disagree
	Intention to reduce the dose after viewing the video	Strongly agree/Agree/Neither agree nor disagree/Disagree/Strongly disagree
	Intention to consult a doctor about reducing the dose after viewing the video	Agree/Neither/Disagree
	Memorable contents	The amount of sleep we need decreases with age./Trying to sleep more than necessary can cause insomnia./Sleeping pills have side effects (e.g., falls, memory loss, etc.)/Hypnotics should be stopped once the cause of insomnia is resolved./Relaxation is the key to sleep./Anxiety about being able to sleep can cause insomnia./Reducing medication can be tricky, so don’t judge yourself. Consult your doctor. /It is natural to have difficulty sleeping immediately after reducing medication./I was sleeping on my own even though I was taking hypnotics

### Analysis of the response of medical professionals to the prototype video

**Fig. 1:**
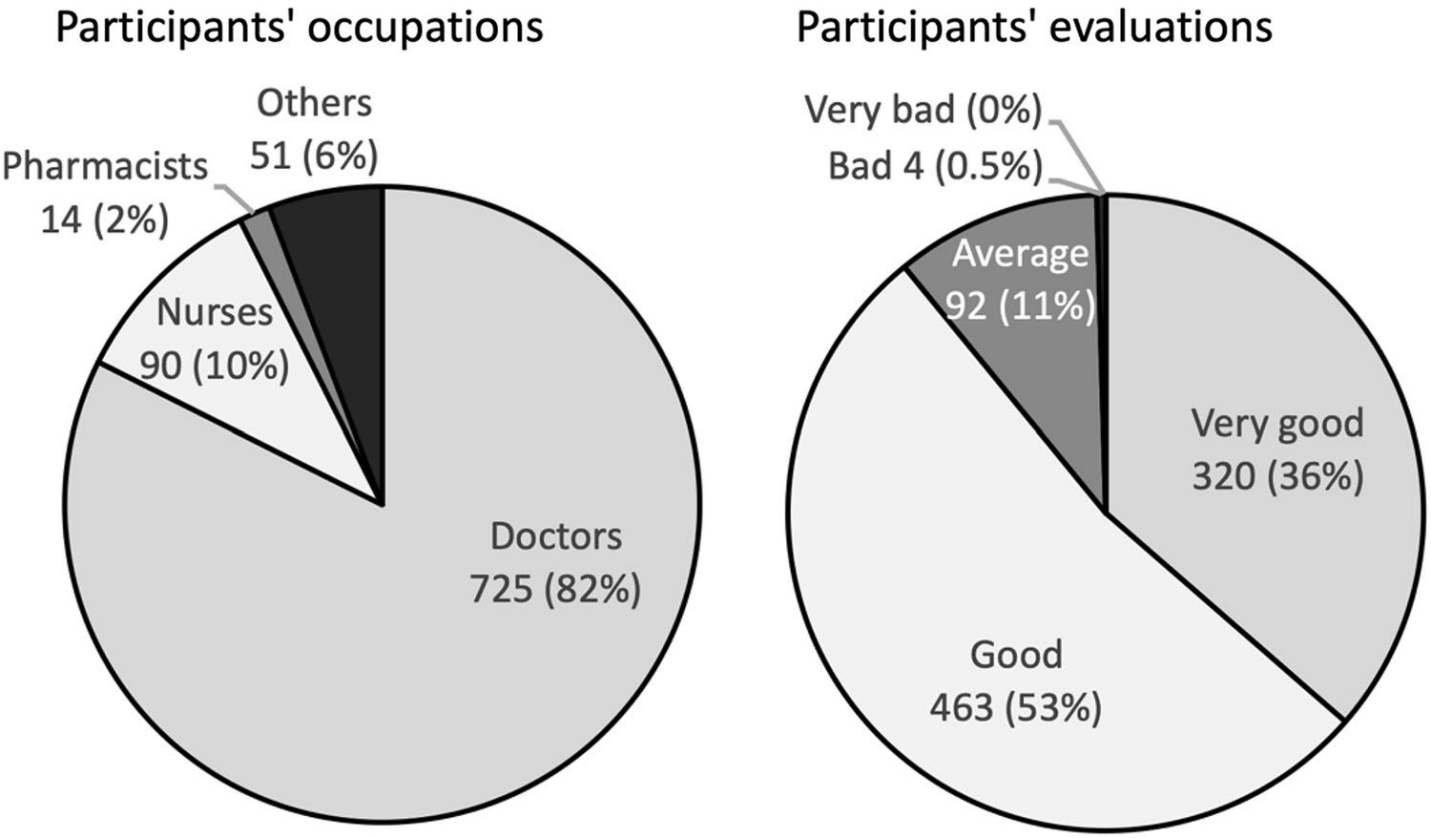
Analysis of the response of medical professionals to the prototype video

Before the public release of the video, the prototype was shown at sleep-related workshops held in various regions of Japan (23 lectures between September 2018 and January 2019). The viewers answered a questionnaire regarding the clinical usefulness of the video and the required modifications (Fig. 1).

### Primary endpoints

Primary endpoints were changes in intentions to reduce medication use before and after viewing the video, and factors associated with improved intention to reduce hypnotic use.

### Statistical analyses

The effectiveness of the video was evaluated by the change in intention of patients to reduce their medication before and after viewing it. Comparisons before and after viewing the video were assessed using the Wilcoxon signed-rank test. The corresponding effect sizes were estimated using the formula *r* = Z/√n, proposed by Rosenthal [18]. For patients who responded to the videos or who ultimately intended to reduce medication, two-group comparisons were performed using the Mann–Whitney *U* test, and the relationship between variables was analyzed using binomial logistic regression analysis. Statistical analysis was performed using SPSS Statistics for Windows (version 22.00) (IBM Software Solutions Japan Inc., Tokyo, Japan). Statistical significance was set at *P* < 0.05.

### Ethics

According to the ethical guidelines for human subject research published by the Japanese Ministry of Health, Labor, and Welfare and the Japanese Ministry of Education, Culture, Sports, Science, and Technology (as revised in 2017) [19], this retrospective study did not require informed consent. Nevertheless, we disclosed information about the study implementation and purpose; documents approved by the Ethics Committee were posted on the website (http://www.jikei.ac.jp/jikei/research_subject/doc/2956/30-347.docx), and posters were displayed in the clinic to guarantee subjects the opportunity to opt-out. Viewers were informed that their data would be used in the study. The survey did not gather any personal information. Participation was voluntary. Viewers were allowed to watch the video without having to complete the questionnaire. Answering the questionnaire was considered consent to participate.

## Results

### Analysis of all respondents

**Fig. 2:**
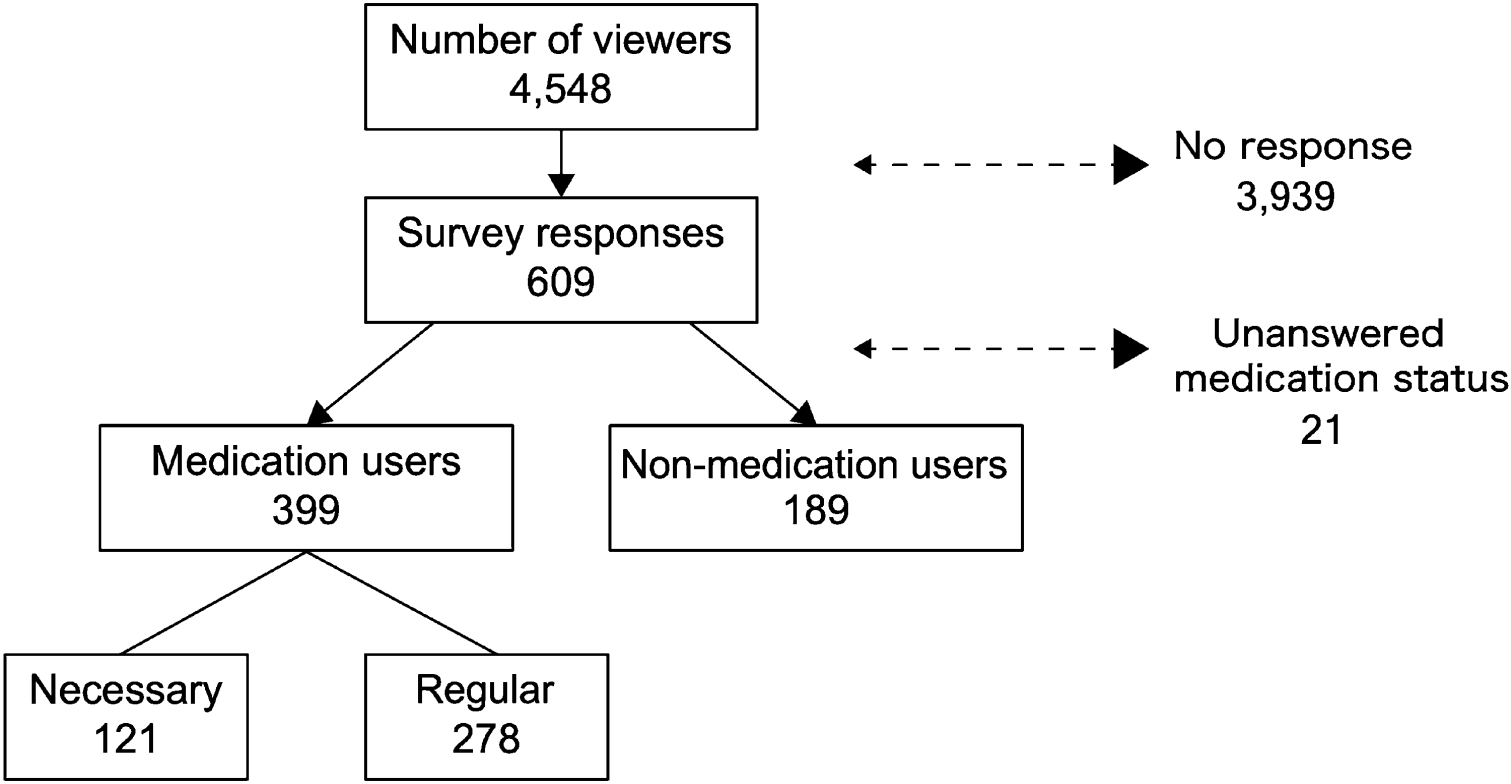
Study flow diagram

In total, there were 4548 viewers, 609 of whom responded to the survey (Fig. 2). The following data were calculated using all of the values entered, including those from questionnaires with incomplete data, with different questionnaires having different missing responses. Each proportion is shown as a percentage of the number of observations excluding the missing values.

There were 275 (46.7%) male and 314 (53.3%) female respondents, with a median age range in the 60 s, distributed with a peak in the 70 s. Two respondents (0.3%) were in their teens, 16 (2.7%) in their 20 s, 56 (9.4%) in their 30 s, 73 (12.3%) in their 40 s, 118 (19.9%) in their 50 s, 113 (19.1%) in their 60 s, 165 (27.8%) in their 70 s, and 50 (8.4%) were over 80 years old.

The reasons for viewing the video were “by chance” 208 (35.2%), “have trouble sleeping” 197 (33.3%), “have anxiety about taking sleep medication” 160 (27.1%), “interested in sleep medicine” 105 (17.8%), “recommendation from acquaintances” 72 (12.2%), “recommendation of my doctor” 46 (7.8%), and “others” 139 (23.5%) (Multiple responses allowed).

Most (95%) of the respondents understood the video; 299 (52.7%) “fully understood,” 242 (42.7%) “understood,” 25 (4.4%) “partially did not understand,” and one (0.2%) “did not understand.” Regarding medication use, 399 respondents (65.5%) indicated that they were taking hypnotics (Online Resource 2; Raw data).

### Detailed analysis of the 399 medication users

There were 185 (47.0%) males and 209 (53.0%) females, with a median age in the 60 s and peak distribution in the 70 s; five patients did not disclose their sex. The frequency of sleep medication use was “daily” for 278 (69.7%) and “only when necessary” for 121 (30.3%) respondents (Table 2).

**Table 2:** Background data

Characteristics	Medication users		Non-medication users	
		Missing values		Missing values
*N*	399	–	189	–
Sex(M/F)	185/209	5	87/101	1
Age(× 10 years)	6.0 ± 1.4	2	4.7 ± 1.5	-
Insomnia score^a^				
Late sleep	2.8 ± 1.1	5	1.6 ± 0.85	2
Mid-sleep	2.9 ± 1.0	5	1.9 ± 1.1	4
Early sleep	2.9 ± 1.1	5	1.9 ± 1.0	2
Daytime dysfunction	2.6 ± 1.1	5	2.0 ± 0.96	5
Medication frequency (regular/occasional)	278/121	–	N/A	–
Reason for viewing the video (multiple responses allowed)				
Recommendation from their doctor	43	–	2	–
Recommendation from someone else	28	–	43	–
Random discovery	159	–	46	–
Insomnia problems	168	–	23	–
Interest in hypnotics	36	–	68	–
Concerns about hypnotics	144	–	11	–
Other	74	–	65	–
Comprehension score^b^	1.6 ± 0.6	19	1.4 ± 0.5	5

^a^Categorized as follows: 1 = non; 2 = mild; 3 = moderate; 4 = severe; 5 = very severe

^b^Categorized as follows: 1 = fully understood; 2 = understood; 3 = partially understood; 4 = hardly understood; 5 = did not understand at all

**Fig. 3:**
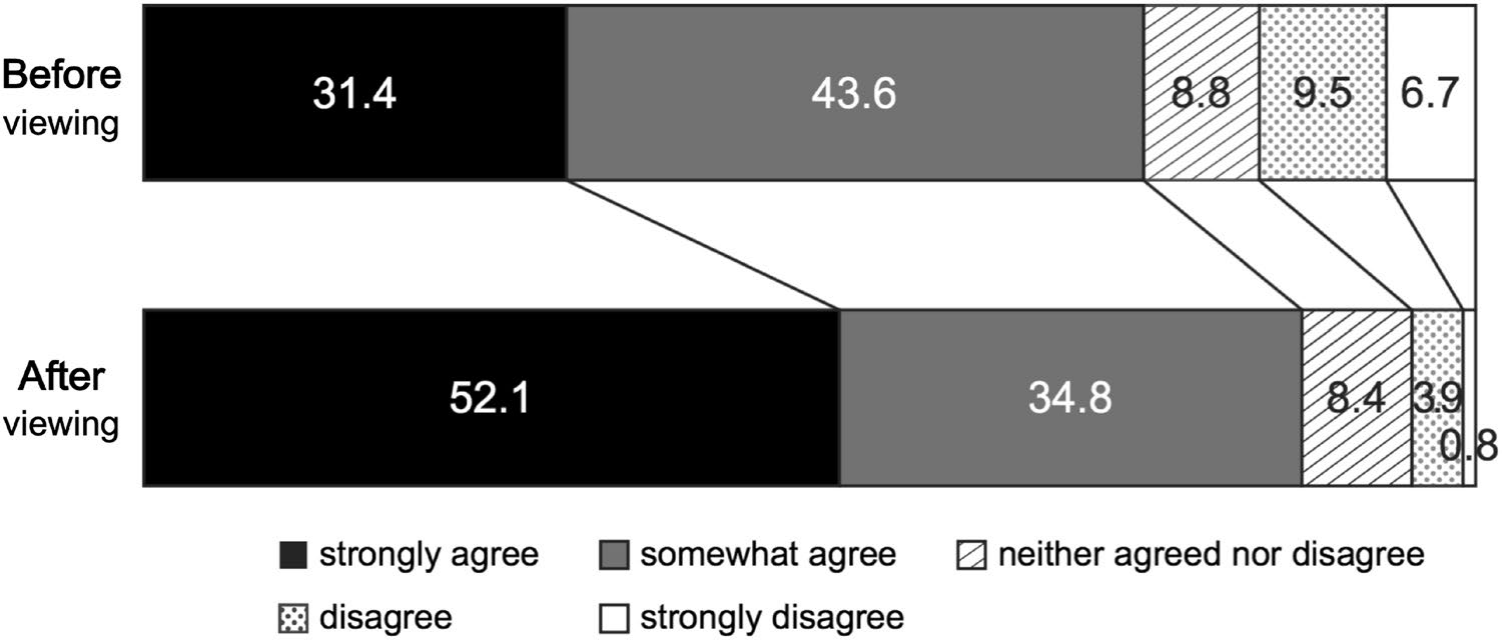
Proportion of respondents with the intention to reduce medication use before and after viewing the video. Before viewing the video, 291/399 (75.0%) medication users intended to reduce medication use. After viewing the video, this increased to 332/399 (86.9%). The intention to reduce medication use significantly improved after viewing the video (*Z* value: − 8.070; effect size: 0.404 [*P* < 0.001])

We measured whether the video changed respondents’ intentions to reduce hypnotics use. Before viewing the video, there were 122 (31.4%) responses of “strongly agree,” 169 (43.6%) “agree,” 34 (8.8%) “neither,” 37 (9.5%) “disagree,” and 26 (6.5%) “strongly disagree.” This indicated that 75.0% of respondents already intended to reduce their medication before viewing the video. After viewing the video, there were 199 (52.1%) responses of “strongly agree,” 133 (34.8%) “agree,” 32 (8.4%) “neither,” 15 (3.9%) “disagree,” and three (0.8%) “strongly disagree.” More respondents [332 (86.9%)] intended to reduce their medication use after viewing the video (Fig. 3).

Additionally, 143 (37.7%) respondents showed stronger intention to reduce medication use, 201 (53.0%) had unchanged intention, and 35 (9.2%) showed weaker intention. The Wilcoxon signed-rank test showed a significant strengthening of abstinence intentions after viewing the video (*Z* value: − 8.070; effect size: 0.404 [*P* < 0.001]).

**Fig. 4:**
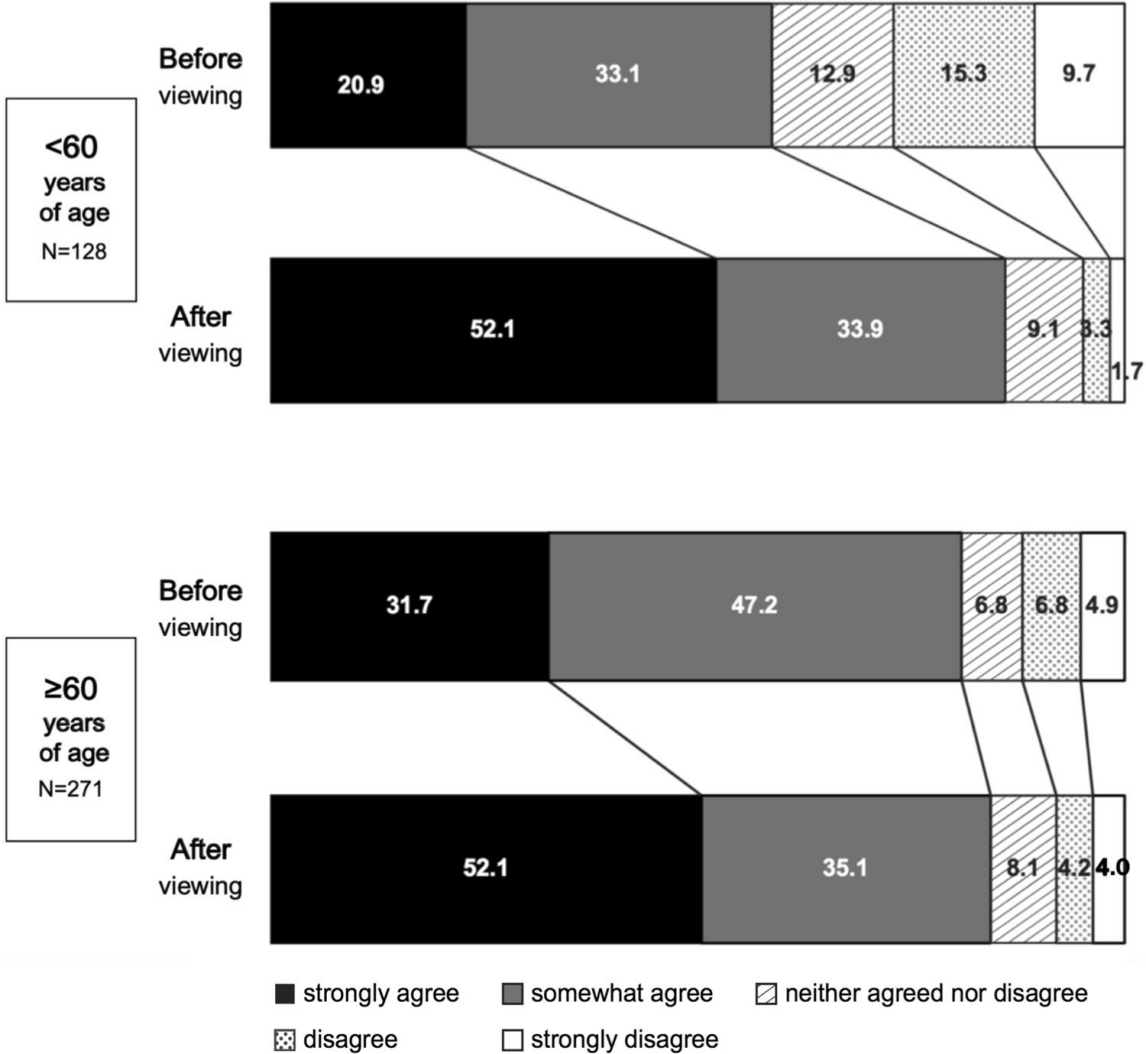
Proportion of respondents with the intention to reduce medication use before and after viewing the video according to age. The intention to reduce medication use significantly improved in both age groups

Considering the background of different mechanisms of insomnia in each generation, the video’s effects were similarly examined in two age groups: < 60 and ≥ 60 years. Both groups had significantly stronger intention to reduce medication use [< 60 years of age: effect size: 0.522 (*P* < 0.001) and ≥ 60 years of age: effect size: 0.340 (*P* < 0.001)] (Fig. 4).

A significant improvement in intention to reduce medication use was observed among 52 (82.5%) participants who initially were averse to reduction, i.e., those who disagreed or strongly disagreed to reduce medication use before they saw the video [*Z* value: − 6.391; effect size: 0.818 (*P* < 0.001)]. Among respondents with no intention to reduce their medication, none became more extreme in their resolve to not reduce their hypnotics use.

After viewing the video, 253 (66.1%) participants responded that they would like to consult their doctor about reducing their medication for various reasons (multiple responses allowed): 189 (75%) did not want to rely on medication, 136 (54.6%) were afraid of the side effects, 66 (26.5%) felt convinced by the video, 33 (13.3%) felt they were taking more medication than necessary, and eight (3.2%) had other reasons. Conversely, 33 (13.0%) respondents admitted that they did not want to consult their doctor after viewing the video. However, 23 (69.7%) of the respondents indicated their intention to reduce their medication. The most common reason given (by 13 respondents, 65.0%) was that they were not comfortable talking to their doctor. Five (25.0%) participants responded that they had no problems taking their medication in the past, four (20.0%) responded that they were worried that they would lose sleep if the medication was reduced, and five (25.0%) had other reasons.

To examine the patients about whom the videos were effective, we divided the medication group into two groups: one group with an increase in the intention to reduce medication use after viewing the video, and one without. Using the Mann–Whitney *U* test, we compared each index before and after viewing the video to identify associated factors. Significant differences were found in the following categories: intention to reduce the dose before viewing the video, motivation for viewing the video (“recommendation from acquaintances,” “by chance,” “have insomnia problems,” and “have anxiety about taking sleep medication”), symptoms of insomnia (“difficulty in falling asleep” and “awakening in the middle of the night”), and memorable content about side effects. Binary logistic regression analysis was used to examine the significant explanatory variables. The following items were extracted: viewing by chance, motivation score before viewing, memorable content about side effects, and daytime symptom score (Table 3).

**Table 3:** Binomial logistic regression analysis of factors associated with increased intentions to reduce medication use

Variable	*β*	SD	Wald	DF	SL	Exp (B)
Age	− 0.03	0.107	0.079	1	0.779	0.97
Sex	0.116	0.282	0.17	1	0.68	1.123
Motivation to view by chance^a^	− 0.66	0.299	4.86	1	0.027*	0.517
Motivation score before viewing the video^b^	1.554	0.183	72.137	1	0.000***	4.73
Memorable content about side effects^c^	0.6	0.287	4.364	1	0.037*	1.822
Presence of insomnia symptoms	0.241	0.159	2.283	1	0.131	1.272
Daytime symptom score^d^	− 0.614	0.241	6.51	1	0.011*	0.541

*β* regression coefficient, *SD* standard deviation, *Wald* wald square, *DF* degrees of freedom, *SL* significance level, *Exp (B)* odds ratio

^*^P < 0.05, **P < 0.01, ***P < 0.001

^a^Answered “by chance” when asked the reason for viewing the video

^b^Intention to reduce medication use before viewing the video. Categorized as follows: 1 = strongly agree; 2 = somewhat agree; 3 = neither agree nor disagree; 4 = disagree; 5 = strongly disagree

^c^Answered “about the side effects of hypnotics” to the question about contents that left a particularly strong impression

^d^Degree of daytime functional impairment (including insomnia symptoms)

Categorized as follows: 1 = not at all; 2 = a little; 3 = somewhat; 4 = very much; 5 = extremely much

We also divided the medication group into those who indicated an intention to reduce medication use after viewing the video and those who did not, regardless of their original intention. In this way, we examined patients who finally have the intention to reduce their medications, including those who were originally willing to do so. Binomial logistic regression analysis was used to examine the significant explanatory variables. Results included comprehension and the memorable content “how hypnotics should be stopped once the cause of insomnia is resolved” (Table 4).

**Table 4:** Binomial logistic regression analysis of factors associated with the intention to reduce medication use after viewing the video

Variable	*β*	SD	Wald	DF	SL	Exp (B)
Age	− 5.987	0.133	0.203	1	0.652	0.942
Sex	0.151	0.396	0.146	1	0.702	1.163
Comprehension score^a^	− 8.935	0.323	7.668	1	0.006**	0.409
Motivation score before viewing the video^b^	0.114	0.296	0.148	1	0.7	1.121
Memorable content about side effects^c^	0.683	0.413	2.728	1	0.099	1.979
Memorable content about how to reduce medication^d^	1.093	0.467	5.481	1	0.019*	2.984

*β* regression coefficient, *SD* standard deviation, *Wald* wald square, *DF* degrees of freedom, *SL* significance level, *Exp (B)* odds ratio

^*^P < 0.05, **P < 0.01, ***P < 0.001

^a^Categorized as follows: 1 = fully understood; 2 = understood; 3 = partially understood; 4 = hardly understood; 5 = did not understand at all

^b^Intention to reduce medication use before viewing the video. Categorized as follows: 1 = strongly agree; 2 = somewhat agree; 3 = neither agree nor disagree; 4 = disagree; 5 = strongly disagree

^c^Answered “about the side effects of hypnotics” to the question about contents that left a particularly strong impression

^d^Answered “hypnotics should be stopped once the cause of insomnia is resolved” to the question about contents that left a particularly strong impression

## Discussion

### Effectiveness of the video

The results indicated that 37.7% of participants had stronger intention to reduce medication after viewing the video, which reflects a statistically significant increase in motivation. Furthermore, the computed effect size of 0.404 suggested a moderate practical significance. Of note, 85.2% of participants who had negative thoughts about reducing their medication before watching the video showed a stronger intention to reduce medication use; the calculated effect size of 0.818 suggested a large practical significance. According to previous studies, single unidirectional psychological education, such as instruction and leaflet distribution, increases the intention to reduce medication use [15, 16]. Our study used a similar intervention.

Although the Internet is perceived to be used mostly by young people, respondent distribution peaked among people in their 70 s, suggesting that dissemination of medical information on the Internet is also effective for older people.

Our video included general sleep hygiene instructions, medication reduction methods, and intentionally included motivation messages that have not been previously published. Although studies suggest that patients’ intentions and motivations are important for successful medication reduction [11, 12], there are no studies targeting motivation. In this video, we referred to Prochaska’s behavior change model [17] as a motivational technique, and used behavior change approaches, such as awareness, emotional experience, self-review, and confidence-building.

In the questionnaire survey, approximately half (49.4%) of the participants who took hypnotics characterized the content of the video as impressive and responded “I was sleeping on my own ability even though I was taking hypnotics”. This suggests that many patients with insomnia lose confidence in their ability to sleep. When cognitive dissonance is induced by the inability to reduce medication, even if one intends to reduce the dose, motivational techniques can be used to resolve ambivalence using a decision balance sheet [20]. In our video, we discussed a potential benefit of this new motivational approach.

### Factors associated with the intention to reduce medication use

We examined factors associated with increased intention to reduce medication use (i.e., variables that increased intention by one or more points after viewing the video) using binomial logistic regression analysis. Four significant explanatory variables were identified: viewing by chance, motivation score before viewing, memorable content about side effects, and daytime symptom score. We expected that the intention to reduce medication use before viewing would be related, and that strong intentions would remain such. The other three significant factors suggest that patients who had been using hypnotics without obtaining appropriate medical information may be more likely to strengthen their intention to reduce medication use. A British survey reported that most elderly patients using benzodiazepines were not warned by their physicians about side effects [21]. Our study also showed that similar trends may exist in primary care in Japan. Additionally, we conducted a binomial logistic regression analysis by dividing the participants into two groups according to their final intention to reduce medication use. Two significant explanatory variables were extracted: comprehension and the memorable content on how hypnotics should be stopped once the cause of insomnia is resolved. Regarding comprehension, a previous study on the reduction of benzodiazepine use [22] reported that this intervention was effective in the absence of cognitive impairment, which is consistent with our results. Moreover, as evidenced by the association between how hypnotics should be stopped once the cause of insomnia is resolved and the intention to reduce the dose, some patients take hypnotics without recognizing the need for dose reduction; therefore, it is possible that merely increasing their knowledge of medication reduction may provide enough motivation. Our results highlight the importance of physicians offering adequate explanations, including of reduction or withdrawal, before prescribing sleep medication [19].

### Consulting a doctor about reducing medication

We found that 66.1% of participants desired to consult their doctor about medication reduction after viewing the video. Conversely, 69.7% of patients who did not wish to consult their doctor intended to reduce their medication, indicating that there was a subgroup who did not consult their doctor about reducing their medication despite their intention to do so. In this small subset (*n* = 23) of respondents, the most common reason was “it is difficult to consult my doctor,” which was cited by 13 patients (more than half). This suggests that physicians may be unintentionally depriving patients of the opportunity to reduce their medications. As one strategy to address this, we propose the active use of Shared Decision Making (SDM); a healthcare delivery model that mandates patient-centered care for clinical practice[23], in sleep medicine as well.

### Practicality of the video

In Japan, where benzodiazepines account for approximately 80% of all hypnotics, the revision of medical reimbursement in April 2018 tightened restrictions on long-term prescriptions and multiple drug usage. However, there is no specific limit for insurance purposes; therefore, limits are dependent on the positive motivation of patients and doctors. Accordingly, we believe that it may be useful to employ educational videos targeting a wide audience. According to the questionnaire for the prototype video, conducted at some sleep-related seminars prior to the release of the video, 89.1% of the 904 medical staff members responded highly to the video. Therefore, we believe that (1) playing the video in waiting areas of hospitals and pharmacies, (2) encouraging patients to watch the video, and (3) creating/disseminating a translated version of the video via social media would be useful in promoting the reduction of sleep medication.

### Limitations

This study has some limitations. The comorbidities, multiple medications, and experience of drug reduction of the participants are unknown. Viewing the video and completing the questionnaire were voluntary actions, and only approximately one in eight viewers responded. It is possible that many of the participants viewing the video were originally positive to reduce medication exists; which could have resulted in selection bias. The responses, including insomnia symptoms and medication status, were self-reported and subject to several important confounders. For example, social desirability bias could have prevented some responders from disclosing excessive hypnotics use. Additionally, the acquiescence bias may have influenced our respondents to choose answers on the left-hand portion of the Likert-type scale. Multiple responses by the same person could not be excluded. Counting views of the video was based on its access count, so views with interruptions were included.. To mitigate these limitations, we need to target a larger number of medication users in medical institutions with a better viewing environment, individualize responses, and check the content of the responses against medical records.

## Conclusions

This is the first study to examine the effectiveness of a short online video on patients’ motivation to reduce or stop sleep medication, and factors associated therein. The results suggest that this intervention increases patients’ motivation to reduce or stop using hypnotics. Moreover, informing patients about side effects and specific ways to reduce their medication use is important to strengthen their intention and promote uneventful medication reduction.

### Supplementary Information

Below is the link to the electronic supplementary material.

Supplementary file1 (PDF 128 KB)

Supplementary file2 (PDF 1157 KB)

## Data Availability

The data that support the findings of this study are available from the corresponding author upon reasonable request.
