# Supplementary material for: Effect of a short video on patients’ motivation for dose reduction or cessation of hypnotics
Source: Sleep Biol Rhythms. 2023 Jan 27;21(3):299–308. doi: 10.1007/s41105-023-00446-4 (PMC10900041; doi:10.1007/s41105-023-00446-4)
Supplement: Supplementary file 1 — Supplementary file1 (PDF 128 KB) [file 41105_2023_446_MOESM1_ESM.pdf]

Online Resource 1; Questionnaire form

Effect of a Short Video on Patients' Motivation for the Dose Reduction or Cessation of Hypnotics

Misato Amagai[a], Motohiro Ozone[b]*, Tomohiro Utsumi[a], Ayana Hotchi[a], Masayuki Iwashita[a], Wataru Yamadera[a], and Masahiro Shigeta[a]

[a]*Department of Psychiatry, The Jikei University School of Medicine, Tokyo, Japan;*
[b]*Department of Neuropsychiatry, Kurume University School of Medicine, Fukuoka, Japan*

*Corresponding author:

Motohiro Ozone, M.D., Ph.D.
Department of Neuropsychiatry, Kurume University School of Medicine, 67 Asahi-machi, Kurume City, Fukuoka Prefecture 830-0011, Japan.
Tel.: +81 942 31 7564; Fax: +81 942 35 6041
Email: ozone_motohiro@med.kurume-u.ac.jp

Translated from Japanese into English

This 10-minute video was created to promote sleep hygiene and encourage people to reduce their dosage of sleep medications. After viewing the video, you will be asked to fill out a short questionnaire. This is voluntary, anonymous, and will only take one to two minutes. We appreciate your time and cooperation.

Please read the following before responding.

About Consent for Participation in Survey Research

This study has been approved by the Ethics Committee of Jikei Medical University. The content of the questionnaire does not contain any personally identifiable information. The results of the questionnaire will be used to improve the video and to provide data for this study. A notice of medical research implementation on the Jikei Medical University website provides details on this study. The results of the study will be used in academic conferences and published papers. When these data are used for other research, the research protocol will be created or modified and undergo ethical review, and the newly identified purpose of use and all other information will be made public. Completing this questionnaire will be regarded as consenting to participate in this study. Furthermore, because this questionnaire is anonymous, it is not possible to correct, delete, or withdraw participation in the study after completing the questionnaire. If you understand and agree with the above, please answer the following questions.

1. Please tell us about yourself.

a. Age □Teens □20s □30s □40s □50s □60s □70s □Over 80
b. Sex □Female □Male
c. Medication Status □Non □Daily □Only When necessary

2. Please tell us about your sleep problems in the last two weeks.

a. Difficulty in falling asleep □None □Mild □Moderate □Severe □Very severe
b. Awakening in the middle of the night □None □Mild □Moderate □Severe □Very Severe
c. Awakening in the early morning □None □Mild □Moderate □Severe □Very severe

d. How much do you think your sleep problems interfere with your daytime? (e.g., daytime fatigue, ability to perform work/daily chores, concentration, memory, mood, etc.) □None □A little □Somewhat □Much □Very much

3. Please tell us about your video viewing.

a. How did you know about this video? *Multiple answers possible*
□Recommendation from my doctor, □Recommendation from acquaintances, □By chance, □Have trouble sleeping, □Interested in sleep medicine, □Have anxiety about taking sleep medication, □Others

b. Did you understand the contents of the video?
□Fully understood, □Understood, □Partially did not understand, □Hardly understand, □Did not understand at all

c. Before viewing this video, did you intend to reduce your sleep medication?
□Strongly agree, □Agree, □Neither agree nor disagree, □Disagree, □Strongly disagree

d. After watching this video, do you intend to reduce your sleep medication?
□Strongly agree, □Agree, □Neither agree nor disagree, □Disagree, □Strongly disagree

e. Do you intend to consult with your doctor about reducing your medication dosage?
□Agree, □Disagree, □Neither

1). Please answer the question only if you answered "agree."
What's the reason for that? *Multiple answers possible*
□Because I'm afraid of the side effects of medication.
□Because I feel I'm taking more medication than I should.
□Because I don't want to rely on medication.
□Because I am interested in reducing medication after watching the video.
□others

2). Please answer the question only if you answered "disagree."
What's the reason for that? *Multiple answers possible*

□Because I worried that they would lose sleep if the medication was reduced.
□Because I had no problems taking their medication in the past.
□Because I am not comfortable talking to their doctor.
□Others

f. Which of the following video contents impressed you particularly?
Multiple answers possible
□The amount of sleep we need decreases with age.
□Trying to sleep more than necessary can cause insomnia.
□Sleeping pills have side effects (e.g., falls, memory loss, etc.)
□Hypnotics should be stopped once the cause of insomnia is resolved.
□Relaxation is the key to sleep.
□Anxiety about being able to sleep can cause insomnia.
□Reducing medication can be tricky, so don't judge yourself. Consult your doctor.
□It is natural to have difficulty sleeping immediately after reducing medication.
□I was sleeping on my own even though I was taking hypnotics.

4. Any additional comments (or questions)

That's all.
Thank you very much for your cooperation.
