# Supplementary material for: Effect of a short video on patients’ motivation for dose reduction or cessation of hypnotics
Source: Sleep Biol Rhythms. 2023 Jan 27;21(3):299–308. doi: 10.1007/s41105-023-00446-4 (PMC10900041; doi:10.1007/s41105-023-00446-4)
Supplement: Supplementary file 2 — Supplementary file2 (PDF 1157 KB) [file 41105_2023_446_MOESM2_ESM.pdf]

Online Resource 2; Raw data

2.1. Conversion of questionnaire responses into data

2.2. Raw data of questionnaire responses

Effect of a Short Video on Patients' Motivation for the Dose Reduction or Cessation of Hypnotics

Misato Amagai[a], Motohiro Ozone[b]*, Tomohiro Utsumi[a], Ayana Hotchi[a], Masayuki Iwashita[a], Wataru Yamadera[a], and Masahiro Shigeta[a]

[a]*Department of Psychiatry, The Jikei University School of Medicine, Tokyo, Japan;*

[b]*Department of Neuropsychiatry, Kurume University School of Medicine, Fukuoka, Japan*

*Corresponding author:

Motohiro Ozone, M.D., Ph.D.
Department of Neuropsychiatry, Kurume University School of Medicine, 67 Asahi-machi, Kurume City, Fukuoka Prefecture 830-0011, Japan.
Tel.: +81 942 31 7564; Fax: +81 942 35 6041
Email: ozone_motohiro@med.kurume-u.ac.jp

2.1. Conversion of questionnaire responses into data

Item (abbreviation in the raw data)

1.a. Age (Age); teens=1, 20s=2, 30s=3, 40s=4, 50s=5, 60s=6, 70s=7, over 80=8
1.b. Sex (Sex); male=1, female=2
1.c. Medication Status (MS); daily=1, only when necessary=2, non=3

2.a. Difficulty in falling asleep (D/FA); non=1, mild=2, moderate=3, severe=4, very severe=5
2.b. Awakening in the middle of the day (A/Mid); non=1, mild=2, moderate=3, severe=4, very severe=5
2.c. Awakening in the early morning (A/EM); non=1, mild=2, moderate=3, severe=4, very severe=5
2.d. Daytime disfunction (DD); non=1, mild=2, moderate=3, severe=4, very severe=5

3.a. The motivations for viewing the video (M/V)
Recommendation from my doctor=1, Recommendation from acquaintances=2, by chance=3, have trouble sleeping=4, interested in sleep medicine=5, have anxiety about taking sleep medication=6, others=7

3.b. Comprehension (C)
fully understood=1, understood=2, partially did not understand=3, hardly understand=4, did not understand at all=5

3.c. Intention to reduce medication before viewing the video (M/BV)
strongly=1, agree=2, neither=3, disagree=4, strongly disagree=5
3.d. Intention to reduce medication after viewing the video (M/AV)
strongly=1, agree=2, neither=3, disagree=4, strongly disagree=5

3.e. Willingness to consult with their doctor about medication reduction (W/C)
agree=1, disagree=2, Neither=3

3.e.1) Reasons for choosing “agreed” (R/A)

Because I'm afraid of the side effects of medication=1, Because I feel I’m taking more medication than I should=2, Because I don’t want to rely on medication=3, Because I am interested in reducing medication after watching the video=4, others=5

3.e.2) Reasons for choosing “disagreed” (R/D)

Because I worried that they would lose sleep if the medication was reduced=1, Because I had no problems taking their medication in the past=2, Because I am not comfortable talking to their doctor=3, Others=4

3.f. Impressive video content (Imp)

The amount of sleep we need decreases with age=1, Trying to sleep more than necessary can cause insomnia=2, Sleeping pills have side effects (e.g., falls, memory loss, etc.) = 3, Hypnotics, should be stopped once the cause of insomnia is resolved =4, Relaxation is the key to sleep=5, Anxiety about being able to sleep can cause insomnia=6, Reducing medication can be tricky, so don't judge yourself. Consult your doctor=7, It is natural to have difficulty sleeping immediately after reducing medication=8, I was sleeping on my own even though I was taking hypnotics =9

2.2. Raw data of questionnaire responses

The following is a list of the 609 questionnaire responses that were received.

ID	Age	Sex	MS	D/FA	A/Mid	A/EM	DD	M/V	C	M/BV	M/AV	W/C	R/A	R/D	Imp
1	3	1	3	1	2	2	3	2	2	4	4	2		4	1
2	3	2	1	3	3	4	2	1	2	3	2	3			1235678
3	7	1	1	3	3	2	1	1	2	2	2	1	3		37
4	3	1	3	2	2	2	4	2	1	4	2	3			8
5	5	1	1	2	3	1	1	2	1	1	1	1	135		349
6	5	1	1	3	3	3	2	1	1	1	1	1	3		89
7	7	1	1	2	3	3	2	1	1	2	2	1	13		1234569
8	3	1	3	1	1	1	1	57	1	4	1	1	4		13478
9	5	1	3	1	1	1	1	2	1	2	1	1	12345		124567
10	4	1	3	1	1	1	1	2	1	3	3	3			9
11	4	1	1	4	4	4	3	1	1	2	2	1	3		249
12	4	2	1	3	4	4	4	1	1	4	1	1	1		3
13	6	2	1	2	3	3	3	1	2	2	2	1	3		289
14	5	1	3	1	2	1	2	7	2	2	2	3			9
15	5	1	2	1	2	2	2	2	1	3	2	1	4		9
16	6	2	1	3	3	3	3	2	2	2	2	3			1489
17	3	1	3	1	1	1	1	35	1		1	1	4		179
18															
19	4	1	3	2	2	1	2	35	1	4	2	3			489
20	3	2	3	1	2	1	1	257	1	1	1	1	5		2469
21	6	1	1	2	2	3	3	1	2	3	2	1	3		1356
22	3	2	3	1	1	1	1	5	1	1	1	1	3		19
23	6	1	2	1	2	2	2	16	2	2	2	1	134		13
24	3	1	2	4	3	4	5	346	1	1	1	1	13		79
25	6	2	3	1	1	1	1	5	1	1	1	1	4		127
26	6	1	1	4	4	4	4	1							
27	3	1	3	1	2	2	2	57	1	1	1	3			89
28	4	1	1	1	1	1	2	1	1	2	2	1	1		1234789
29	5	2	1	4	4	4	3	146	1	1	1	1	124		123468
30	3	1	3	1	2	1	2	567	2	5	5	3			268
31	4	2	2	2	1	1	1	2	1	5	5	3			123
32	6	1	1	3	3	3	3	1	1	2	2	1	14		79
33	3	1	3	2	2	2	1	2	1	2	1	3			4
34	4	1	1	3	2	1	4	1	1	2	1	1	134		1345789
35	5	1	1	3	4	3	3	2	1	2	2	2		2	15
36	5	1	2	2	3	1	4	1	2	3	4	2		4	12347
37	4	1	2	1	1	1	2	27	1	2	1	1	13		136
38	4	1	1	4	2	2	4	14	1	2	2	1	3		2389

39	2	2	3	2	2	1	2	2	2	3	2	1	4		2679
40	3	1	3	1	1	1	1	3	1	1	1	1	1		5
41	3	2	1	5	2	1	3	14	1	4	2	1	134		9
42	3	2	1	3	3	4	3	2	2	2	2	3			123
43	4	2	1	3	4	4	4	3	2	1	2	1	3		12489
44	5	1	1	3	1	1	2	1	1	2	1	1	1		3478
45	6		2	3	3	3	2	1	2	1		1	3		12345678
46	5	1	2	2	1	2	2	1	1	3	1	1	3		29
47	5	1	3	2	2	2	2	3	1	1	1	1	3		4
48	6	1	1	3	3	2	2	6		2		2		4	49
49	4	2	3	1	1	1	2	7	2						9
50	4	1	2	2	3	4	2	3	1	3	3	3			1268
51	4	1	3	1	1	2	2	5	1	1	1	1	4		149
52	4	2	3	1	1	1	1	27	1						489
53	4	2	3	3	3	2	2	25	1						1
54	4	1	3	2	3	3	3	7	1	3	3	3			29
55	4	1	3	1	1	1		2	1	3	3	3			27
56	7	1	1	4	3	3	3	1	3	5	2	1	3		1349
57	3	2	1	4	3	2	3	346	1	1	1	1	1		1479
58	4	2	3	1	1	1	1	5	1	5	5	3			2
59	2	2	3	1	1	1	1	7	1	1	1	3			23479
60	3	2	3	1	2	1	1	2	1						89
61	7	2	1	4	4	4	3	7	2	2	2	2		3	6
62	7	2	1	1	1	1	1	1	2	2	1	1	3		1
63	5	2	1	2	2	2	2	456	1	2	1	1	1234		234
64	5	2	3	1	1	1	1	2	1						1
65	4	2	3	1	1	1	1	7	2	3	3	3			239
66	3	1	2	2	1	2	1	7	2	2	1	1	3		139
67	5	2	1	2	1	1	3	4	3	2	1	1	3		7
68	4	1	3	1	2	2	1	5	1	5	1	1	1		1
69	5	1	3	1	1	1	2	2	1	4	2	1	2		4
70	5	1	3	1		2	1	5	2	1	1	3			1236
71	5	2	1	3	3	2	3	1	1	5	2	1	134		139
72	2	2	2	3	3	3	3	3	2	5	2	1	13		13
73	3	1	3	1	1	1	2	5	1	1	1	3			9
74	5	2	3	1	1	2	1	5	2	3	1	3			12
75	4	1	3	1	1	2	2	7	1	2	2	1	4		12
76	5	2	1	2	3	4	3	7	1	2	1	1	1345		13478
77	5	1	3	1	1	1	1	2	1	5	1	3			4
78	7	1	1	4	5	4	3	14	2	5	4	1	4		1246
79	7	1	1	3	3	4	3	146	1	4	1	1	134		12345678
80	5	1	3	1	1	3	1	2	3	5	5	2		4	59
81	5		1	3	3	3	2	2	3	5	3	1	5		1
82	5	1	1	2	3	3	3	12	3	5	1	1	2		1247
83	5	1	3	2	1	1	1	2	2	5	5	3			12
84	3	2	1	3	3	2	1	26	2	2	3	1	4		14

85	6	1	1	4	2	3	2	12	2	5	2	1	4		13467
86	4	1	2	1	1	1	2	2	2	5	3	2		2	12368
87	6	2	1	1	1	1	1	2	2	5	5	3			
88	6	2	1	3	3	3	2	2	3						
89	5	1	1	2	2	2	2	24	1	2	1	1	24		157
90	6	1	3	1	1	1	1	5	1	1	1	1	12		12345678
91	5	1	2	5	5	5	3	3	1	1	1	1	13		12345678
92	4	2	3	1	2	1	3	57	1	1	1	1	4		1589
93	5	1	1	3	4	4	3	346	2	2	1	1	13		12456789
94	7	2	2	4	3	3	1	146	2	1	3	1	13		1256
95	5		3	1	1	2	1	7	1	1	1	3			
96	4	2	3	1	2	2	1	27	2	5	2	3			149
97	3	2	3					7	1						1
98	3	2		3	3	3	4	34	2	4	2	3			379
99	2	2	3	2	2	2	2	2	2	1	1	1	13		49
100	4	2	3	1	1	1	2	25	1	1	1				12369
101	2	2	3	2	2	2	4	34	2	1	1	1	13		23456789
102	4	2	3	1	2	1	3	7	2			3			123489
103	7	2	2	2	3	2	1	14	1		1				129
104	7	1	1	2	2	3	5	34	1	2	1	3			1245689
105	4	2	1	1	1	2	4	3	2	4	2	3			12345678
106				5	4	4	4	46	2	2	2	1	124		234689
107	4	2	3	2	2	1	2	7	1						2
108	6	1	1	1	1	4	3	1	2	2	2	1	3		129
109	6	2	1	3	1	1	1	3	1	2	1	1	3		489
110	6	2	3	1	1	1	3	5	2	2	1	1	4		12349
111	5	2	3	1	2	1	1	7	1						3789
112	3	2	1	3	1	4	4	1	1	4	1	1	1234		5689
113	5	1	1	3	3	3	3	2	2	4	2	3			1256
114	7	1	1	3	3	3	4	1	2	4	1	1	124		12379
115	5	2	1	3	4	4	4	346	2	2	2	3			1234579
116	2	1	3	3	4	4	3	2	1	2	1	3			26
117	3	2	1	2	2	2	3	34	2	4	2	1	3		34
118	4	1	3	1	1	1		57	2	1	1	3			48
119	5	1	3	2	3	3	3	2	1	3	2	3			123679
120	6	1	1	2	3	4	2	1	2	2	2	1	13		124789
121	7	2	2	2	1	2	2	25	2	3	2	2		3	12345678
122	7	2	2	2	2	2									
123	7	2	1	1	1	1	1	1	2	1	1	1	134		1378
124	7	1	3	1	1	1	1	3	2	3	3	3			123456
125	7	1	2	2	4	3	2	7	1	1	1	1	13		139
126	8	2	1	3	4	4	2	46	2	3	2	3			34
127	5	2	1	3	3	3	2	7	1	2	1	1	3		39
128	7	1	1	3	4	4	1	3	1	2					4
129	6	1	3	2	2	2	1	47	1	3	2	3			1239
130	7	1	2	1	2	3	2	3	1	2	2	1	3		2345

131	7	2	2	4	3	4	3	3	1	2	1	1	1		369
132	5	2	3	1	1	1	2	67	1						6
133	5	2	2	1	3	2	2	4		2	2	2		4	3
134	4	2	3	1	1	1	1	5	2	1	2	3			3489
135	4	1	3	1	1	1	1	7	1	2	2	1	4		12345678
136	7	1	1	3	3	4	4	46	2	2	1	3			349
137	6	1	1	2	3	3	3	4	2	3	2	1	13		269
138	6	2	3	1	2	3	2	3	1	3	3	3			12
139	7	2	3	2	1	1	2	3	2						345
140	8	2	1	2	4	3	3	346	2	4	2	3			2379
141	6	2	2	4	4	2	3	3456	1	1	1	1	3		2569
142	5	2	1	2	3	3	3	47	1	2	1	1	1		348
143	5	2	1	1	2	4	2	36	2	2	1	3			23489
144	6	2	3	2	4	4	3	46	1						12569
145	6	2	1	4	3	1	2	346	1	1	1	1	13		2369
146	5	2	2	3	3	1	3	4567	2	4	1	1	134		12345689
147	7	2	1	3	3	3	2	4	2	2	2	3			378
148	7	1	3	3	3	3	2	345		5		3			15
149	8	1	1	1	1	1	1	3	1	2	2	3			15689
150	5	2	3	1	1	1	1	7	1	1	1	3			18
151	5	2	1	2	2	2	1	6	1	1	1	1	13		489
152	8	2	2	2	3	3	2	2	2	1	2	2		4	25
153	8	1	1	3	3	3	1	7	2	3	1	1	1		149
154	6	2	1	5	2	2	3	7	1	1	1	1	3		2789
155	4	2	3	2	2	1	1	5	1	1	1	1	13		9
156	8	1	2	3	3	3	2	46	2	2	2	3			34789
157	7	1	3	1	5	4	2	347	2	3	2	3			25678
158	5	2	2	3	2	3	2	3							
159	7	2	1	1	3	3	3	4	1	1	1	1	3		245689
160	6	2	1	2	3	2	3	46	1	2	1	1	34		12345678
161	7	2	1	5	5	5	3	346	2	2	2	3			13469
162	6	1	1	1	4	4	2	3456	1	2	1	1	3		1489
163	7	1	1	3	3	1	4	6	2	2	2	1	1		13469
164	7	1	2	3	4	4	3	346	2	2	1	2		3	1269
165	7	1	3	2	5	4	5	47	2	1	1	1	13		34689
166	8	1	1	1	3	4	5	47	2	4	4	2		1	123
167															
168	8	1	1	3	4	3	4	4	2	1	2	1	134		123479
169	6	2	2	2	2	2	2	356	1	1	1	3			134589
170	6	2	2	3	3	2	3	7	1	2	1	3			123489
171	7	2	1	4	2	1	3	346	2	1	1	1	3		256
172	5	2	1	3	3	3	2	46	2	1	1	1	3		12369
173	4	2	3	1	1	1	1	5	1						6789
174	7	2	1	3	2	2	2	46	1	1	1	1	123		12346
175								46							
176	7	1	1	4	2	1	1	7	2	2	1				249

177	7	1	2	2	2	2	2	6	1	3	1	1	1		12345678
178	6	1	3	3	4	4	2	7	2						
179	6	2	2	3	2	1	2	7	1	1	1	1	3		39
180	7	1	2	1	2	2	1	34	2	1	1	1	1		13
181	6	2	1	4	1	1	1	4	1	2	2	3			8
182	7	2	1	2	4	4	3	346	1	1	1	1	13		49
183	5	2	1	2	2	1	3	36	1	2	1	3			13459
184						2									
185	7	2	1	4	4	3	4	467	2	1	1	1	14		345789
186	7	1	3	1	3	3	3	36	2						12
187	8	1	1	4	3	4	4	3	2	1	1	1	1		12345678
188	6	1	1	3	3	4	4	47	1	2	1	1	124		23489
189	7	1	3	2	2	1	1	3	1						1
190	8	1	2	3	3	3	3	4	2	2	2	1	3		124569
191	8	1	3	2	2	3	3	457	2						5
192	7	1	3	1	3	3	3	34	1						25
193	8	1	1	2	3	3	4	6	3	1	1	1	134		237
194	6	2	2	4	4	2	2	3	2	2	2	2		2	12
195	6	2	1	1	2	2	3	6	3	2	2	1	4		3489
196	8	1	2	4	2	4	3	46		2	2	1	1		23
197	6	1	1	1	3	3	3	3	2	2	2	1	3		1479
198	6	1	1	3	3	2	3	36	1	2	2	1	13		129
199	7	2	1	2	3	3	3	346	2	2	2	1	34		345678
200	6	2	2	2	2	1	1	6	1	1	1	3			89
201	6	2	1	4	5	5	3	6	1	1	2	2			9
202	7	1	1	2	2	2	2	356	3	2		3			168
203	7	1	1	2	3	4	3	4	2	2	2	1	13		2489
204	7	1	2	1	3	3	3	4	2	2	1	3			145789
205	7	1	2	3	3	3	3	3	2	3	3	3			13
206	7	2	2	2	3	2	3	7	1	2	1	1	3		345689
207	2	1	2	4	3	2	2	7	1	3	3	1	3		1
208	7	2	3	3	3	3	2	57	2						2356
209	8	1	3	1	1	1	3	35	2	1	1	1	3		5
210	8	1	1	3	3	3	3	6	2	5	2	3			12345678
211	6	1	1												
212	7	1	2			3	3	7	1	2	1	1	13		69
213	7	1	3	2	3	3	3	34		3	3				1257
214	7	1	3	2	1	1	1	3	2	1	1	3			5
215	7	1	1	3	3	4	2	46	1	1	1	1	13		168
216	6	2	2	4	3	2	2	356	1	1	1	1	34		12689
217	8	1	1	1	1	1	1	3	1	3	3	3			135789
218	8	2	2	2		2	2	7	2	2	2	3			26
219	7	2	1	2	2	3	1	46	2	2	2	1	34		1234679
220	6	2	1	4	4	4	2	4	2	3	1	1	24		1345
221	8	1	1	3	3	2	1	6	1	2	1	3			236
222	5	2	3	4	3	1	3	7	1	3	2	3			349

223	7	2	1	4	3	2	3	46	2	1	1	1	13		2469
224	5	1	3	1	3	3	2	7	1	3	1	3			18
225	7	2	1	4	3	3	4	7	2	2	1	3			46
226	7	2	1	5	4	4	4	46	1	1	2	3			2789
227	6	2	1	4	4	5	4	47	1	3	3	1	3		69
228	8	1	1	3	3	3	4	34	3	2	2	1	13		1245678
229	6	1	1	3	3	4	3	46	2	2	2	1	13		148
230	7	2	1	3	4	2	1	3	1	1	1	1	1		2469
231	7	1	1	2	3	3	3	3	2	2	2	3			2345789
232	8	1	1	3		3	3	356		2		3			
233	6	2	1	3	2	3	2	6	2	1	1	1	3		48
234	7	2	1	4	4	4	2	346	1	1	1	1	13		379
235				3	3		4	3456		2	2	1	3		12356
236	7	1	1	2	4	5	4	3456	1	1	1	1	13		12345678
237	6	2	2	2	2	2	2	47	1	2	1	3			12369
238	8	1	2	3	3	3	2	3	2	2	1				12345678
239	7	1	1	2	2	2	2	47	2	2	2	3			13469
240	7	1	1	4	5	5	5	3	2	4	4	3			14589
241	8	1	2	4	4	3	2	346	3	2		1	134		269
242	6	2	2	3	3	3	2	346	2	1	2	1	13		24789
243	7	2	1	4	4	4	3	34	1	2	1	1	13		35678
244	8	2	3	1	1	2	1	3	1	3	3				5
245	6	2	2	2	1	1	1	34	1	3	1	1	13		123689
246	8	1	1	4	3	4	4	36	2	1	2	1	135		234678
247	7	1	1	2	3	4	1	4	1	2	1	1	3		12345678
248	7	1	1	2	3	4	1	4	1	2	1	1	3		12345678
249				1	1	5		16				2		3	345689
250	6	1	1	3	4	4	2	7	1	5	2	1	4		37
251	7	1	1	3	2	3	2	346	1	1	1	1	134		2369
252	7	2	3			2	2	6	1		1				1235
253	7	2	2	3	3	2	3	6	2	1	1	3			23568
254	7	1	1	4	4	3	3	3							
255	7	1	1	4	4	1	2	3	2	2	2	3			12345678
256	7	1	1	3	3	3	2	4	1	1	1	1	1		12458
257	6	1	3	2	3	3	2	47	1	1	1	3			1234569
258	7	2	2	1	3	2	1	36	3	2	4				9
259	7	1	1	5	4	4	1	134	2	1	2	1	1		123467
260	7	1	1	5	4	4	1	134	2	1	2	1	1		123467
261	7	2	1	4	4	5	4	346	2	1	1	1	13		489
262	5	1	1	3	3	4	4	7	2	1	1	1	13		89
263	7	1	2	2	2	3	3	346	1	1	1	3			24569
264	5	2	3	1	2	2	1	357	1	3	3	3			9
265	7	2	3	3	4	4	1	6	3						1378
266	6	2	2	2	2	2	2	3	1	2	1	1	1		13569
267	7	1	2	1	3	4	3	3	2	3	3	3			49
268	6	1	3	2	2	1	3	5	1	3	3	2		4	1235679

269	6	2	1	1	4	4	3	4	2	4	3	3			3
270	7	2	1	2	4	4	4	3456	2	2	2	1	13		
271	7	2	1	5	4	4	2	2	2	5	2	3			1356789
272	7	1	1	3	3	3	2	46		2	2	3			347
273	7	1	3	2	2	2	2	6	2	2	1	3			14
274	7	2	1	2	2	3	1	6	1	5	1	2			12
275	5	2	3	1	1	1	2	7	1	5	1	3			19
276	7	2	1	2	2	3	3	3	1	2	2	3			89
277	7	1	3	1	1	1	1	357	1	5	1				134678
278	7	2	2	3	3	2	1	46	2	3	2	3			139
279	7	1													
280	7	2	2	4	4	4	3	3	2	1	1	1	13		126
281	8	1	1	3	3	4	4	7	2	1	1	1	3		24
282	7	2	2	3	2	1	2	7	1	2	1	1	134		1239
283	6	2	2	3	2	3	2	467	1	2	1	1	34		124689
284	6	2	2	4	3	3	4	4567	1	1	1	1	134		12345678
285	5	2	3	2	4	4	3	34	1	1	1	1	1		1268
286	6	1	3	3	3	4	4	7	2						135
287	5	1	1	3	3	4	1	7	1	2	1	1	23		125
288	7	2	3	2	3	3	3	7	2	1	2	2		4	1234
289	6	2	1	4	3	3	2	46	2	2	1	3			3789
290	6	2	1	2	3	2	1	3	2	1	3	3			
291	7	2	2	3	2	3	2	346	2	2	2	1	3		1489
292	6	2	2	2	4	4	2	46	2	1	1	1	13		13
293	7	2	1	4	4	5	2	16	2	2	1	3			4
294	6	1	1	2	2	2	1	67	1	2	2	3			1489
295	4	2	3	1	2	1	2	7	2	3	3	3			349
296	6	2	3	1	1	2	2	34	2						125
297	7	1	1	4	3	2	1	36	1	2	2	1	13		126
298	5	2	3	2	3	3	3	4	2						9
299	3	2	3	1	1	1	1	5	1	5	5				489
300	7	2	2	3	3	3	2	46	1	1	1	3			1256
301	8	2	1	2	2	2	2	346	1	1	1	2		23	14
302	6	1	2	3	3	3	3	7	2	2	1	1	13		135
303	5	2	2	2	1	1	2	7	1	4	1	1	3		2
304	7	2	1	3	3	3	2	7	2	1	2	1	3		49
305	7	1	1	2	3	2	2	456	1	1	1	1	34		13789
306	7	1	1	2	3	2	2	456	1	1	1	1	34		13789
307	7	2	2	2	4	5	1	46	2	1	3				
308	5	2	1	1	2	2	2	7	2	4	2	1	3		3
309	6	2	1	4	4	1	4	346	1	2	1	1	134		9
310	7	2	2	5	3	1	2	467	2	1	2	1	134		134689
311	7	1	2	1	2	3	1	3	1	2	2	3			7
312	5	2	3	1	3	2	2	47	1						13789
313	6		2	4	4	3	3	3	2	1	1	1	1		4567
314															

315	6	2	1	4	1	1	1	46	1	2	1	1	34		12345689
316	7	1	3	2	3	3	2	5	2	3	3	3			125
317	7	1	3	2	4	3	3	34	1						239
318	8	2	1	3	3	5	4	4	3	5	4	3			49
319	7	2	1	4	3	3	1	467	1	1	1	1	1234		125678
320	6	1	1	4	2	2	2	4	2	2	1	1	13		2346
321	7	1	1	5	5	5	5	3		1	1	1	1		69
322	7	2	1	4	4	4	3	6	2	2	4	1	13		136
323	7	1	1	5	5	4	3	7	1	4	1	1	34		12468
324	7	1	2	2	2	2	2	3	1	2	2	3			1239
325	5	2	3	4	4	3	2	5	2	5	4	3			126
326	5	2	3	4	4	3	2	5	2	5	4	3			126
327	6	2	2	4	2	2	2	46	1	2	1	3			123
328	7	2	2	2	3		1	7	2	2	2	1	3		6
329	5	2	1	5	3	4	2	6	1	2	1	1	13		12345678
330	7	2	2	3	1	1	2	34	2	2	2	3			24569
331	8	1	2	3	3	3	4	346	3	3	3	3			15
332	8	2		3											
333															
334	6	2	2	3	3	3	2	36	1	2	2	1	13		36
335	7	2	1	5	5	5	4	467	1	1	1	1	123		379
336	6	1	2	4	3	3	2	7	3	2	2	1	3		15
337	7	2	2	1	2	2	2	7	1	1	1	3			12345678
338	6	2	2	3	3	3	2	47	2	2	1	2		13	12369
339	6	1	1	2	2	2	2	6	2	2	1	1	123		346789
340	7	2	3	3	3	4	3	4	1						12469
341	6	1	1	4	4	4	3	3	1	2	1	1	3		29
342	7	1	1	3	3	3	4	346	2	2	1	1	13		123456
343	8		1	4	3	3	4	3	1	1	1	1	1		124568
344	5	2	2	2	2	1	1	34	1	4	4	2		123	9
345	7	2	2	1	3	5	3	7	1	4	2	1	4		689
346	6	2	1	3	1	1	1	6	2	2	1	1	34		1235689
347	8	1	1	5	4	3	2	37	1	1	1	1	135		3478
348	8	1	1	5	4	3	2	37	1	1	1	1	135		3478
349	5	2	1	3	3	4	2	3	2	1	1	1	3		3489
350	6	1	1	3	3	1	3	47	2	2	1	1	34		37
351	7	2	1		4	2	3	4	2	2	2	1	3		6
352	4	2	1	5	1	1	5	46	1	1	1	1	123		39
353	8	1	2	4	5	5	3	346	2	2	1	1	34		246
354	4	2	3	3	4	4	4	1	2	3	3	3			56
355	8	2	1	3	2	3	1	6		1	1	1	3		6
356															
357															
358	7	1	1	3	3	2	3	34	1	2	2	1	14		12367
359	7	2	1	4	2	1	1	3456	1	1	1	1	13		23456789
360	3	1	3	1	2	1	2	5	1	5	5	3			12

361	7	2	1	2	3	2	3	345	1	1	1	1	134		89
362	8	2	1	4	4	3	2	456	1	2	1	1	13		3469
363	7	2	2	2	2	2	2	5	2	1	1	3			1569
364	8	2	1	2	3	3	2	3	1	2	2	3			4789
365	6	1	1	4	3	3	4	1	2	2	4	3			1235
366	7	1	3	3	4	3	3	7	2		1				3
367	8	1	3	3	4	3	2	57	2						136
368	6	2	3	2	1	2	1	357	1	2	2	1	14		12347
369	7	1	1	3	4	4	2	46	2	1	2	2		3	346
370	6	1	1	3	1	1	1	7	2	1	2	1	23		124679
371	7	2	1	2	3	4	3	2	2	4	3	1	3		8
372	7	2	1	2	3	4	3	2	2	4	3	1	3		8
373	7	2	1	2	5	5	2	4	1	3	2	3			239
374	8	1	1	2	1	1	1	35	2	3	2	3			13678
375	8	2	1	3	3	3	2	7	1	4	2	3			145789
376	8	1	1	2	2	2		4			1	1			19
377	4	2	3	2	2	2	4	57	1						12349
378	5	2	3	1	1	1	2	7	2	5	2	3			89
379	2	2	3	1	1	1	2	3		2	1	3			1
380	6	2	2	2	2	2	3	46	2	1	1	3			12569
381	7	2	2	3	3	4	3	47	2	2	1	1	3		2469
382	7	1	1	3	3	3	2	47	2	2	2	2		12	136
383	6	2	1	4	4	4	1	346	2	1	1	1	13		124689
384	7	1	1	2	4	4	3	47	1	2	3	1	34		289
385	6	1	3	1	1	1	1	3	1	3	3	3			189
386	7	2	2	3	4	1	3	346	1	1	1	2		13	169
387	5	1	1	4	4	4	3	456	1	1	2	3			269
388	5	2	3	2	1	1	1	5	2						23
389	7	1	3	2	4	4	3	4							
390	6	2	2	1	2	3	3	7	2	1	1	1	13		129
391			2	2	2	2	4	57	1		1	1	13		19
392	7	1	1	3	2	2	3	7	2	2	2	1	123		356789
393	7	2	3	3	3	3	2	4	2	2	1	3			15
394	6	2	3	1	1	1	2	37	1						1289
395	3	1	3	1	1	2	1	7	1	3	3	3			123456
396	7	1		4	4	3	3	34		2	3	3			18
397	8	1	1	2	3		2	7	2	2	3	1			3
398	7	1	1	3	3	3	3	34	2	2	2	1	4		2345679
399	4	2	3	1	1	1	1	7	1						12346789
400	6	2	1	4	3	3	3	3	1	2	2	1	3		1345
401	7	1	2	3	3	2	3	346	1	4	1	1	1234		125
402	7	2	2	1	4	4	2	46	2	2	2	1	3		34
403	4	2	1	2	3	2	3	5	1	4	2	1	4		248
404	7	1	2	2	2	2	2	4	2	3	4	1	3		39
405	6	2	3	1	1	2	1	57	1						14568
406	5	2	1	2	3	3	3	2	1	1	1	1	13		12345678

407	5	2	1	1	1	2	2	2	2	4	2	3			34
408	7	2	3	4	5	3	4	5	1	1	1	1	1		1
409	7	2	1	3	3	4	3	4	3	2	3	1	3		569
410	7	2	1	3	3	4	3	4	3	2	3	1	3		569
411	7	2	1	2	3	3	2	3	1	4	1	3			389
412	3	2	1	3	5	5	1	37	1	4	1	1	234		13
413	7	2	1	4	4	4	1	34	1	2	2	3			2469
414	6	2	3	3	5	5	3	34	1						123569
415	4	2	3	1	1	1	1	7	1	1	1	1	2		2
416	7	2	3	2	2		1	5	1						49
417	8	1	1	5	4	3	3	346	1	1	1	1	13		1349
418	3	2	1	2	2	2	4	346	2	1	1	1	23		1389
419	6	2	1	4	4	4	2	4	1	2	1	1	3		79
420	7	2	1	4	4	3	1	34	2	2	2				2689
421															
422	5	2													
423	5	2	3	1	1	1	1	5	1						89
424	6	2			2	2	2	246	1	1	1	1	34		89
425	6	2	1	3	3	3	3	7	2	2	2				13489
426	5	2	3	1	1	1	3	25	1	1	1	3			129
427															
428	3	2	1	4	4	5	5	37		5		2		13	2
429	5	2	3	1	1	1	1	25	1	2	1	3			135
430	7	1	3	2	3	4	4	7	2	2	1	1	34		16
431	8	1	1	2	2	3	2	36	1	2	1	2		34	1245689
432	4	1	1	3	2	3	2	3	2	5		3			12345
433	7	1	2	2	2	2	2	5	2	1	1	1	4		1234
434	8	2	1	1	3	3	4	3	2	4	4	3			38
435	5	2	1	4	4	5	4	3	3	3	3	1	1		5
436	6	1	3	1	2	2	2	7	2						1246
437	1	2	3	1	1	1	2	3	1	3	3	3			2
438	4	1	3	1	1	1	3	26	1	2	2	3			258
439	4	1	1	1	3	1	1	1	2	2	2	2		1	15
440	7	2	1	2	1	1	2	6	1	2	1	1	13		346789
441	4	1	2	1	2	3	3	2	1	3	1	3			347
442	5	1	3	1	1	2	1	25	1	3	3	3			29
443	4	1	3	1	1	1	1	5	2	2	2	3			9
444	5	1	3	1	1	1	1	25	1	3	3	3			34
445	5	1	3	2	2	3	3	2	2	5	2	1	3		148
446	3	1	3	1	2	2	3	5	1	3	3	3			2
447	4	2	3	1	1	1	4	7	1	3	3	2		4	4
448	4	1	3	1	1	1	1	257	1	1	1	1	4		189
449	3	2	1	3	3	1	5	7	3	5	5	3			124678
450	3	2	3	1	1	1	1	2	1	1	1	3			8
451	6	2	3	1	1	1	2	2	2	5	1	1	3		134
452	5	2	3	1	1	1	2	5	1	1	1	1	4		4

453	6	1	1	4	4	4	3	3	3	4	4	2		1	126
454	4	1	3	1	1	2	3	25	1	5	1	3			469
455	3	2	3	3	3	3	3	2	1	1	1	1	13		1235679
456	5	2	3	4		2	3	7	1						8
457	3	2	2												
458	8	1													
459	8	1	2	1	3	4	3	45	2	2	2	3			479
460	4	2	3	1	2	1	2	5	1	5	3				147
461	2	1	1	2	2	2	3	2	3	3	1	1	2		2
462	7	2	1	2	1	3	2	4	3	1	1	1	3		347
463	7	1	2	3	4	4	3	346	2	2	3	3			3
464	8	2	1	4	4	4	4	4	2	2	1	1	1		12389
465	3	1	3	1	1	3	3	5	2	2	1	3			14
466	5	2	1	2	3	3	2	3	1	5	2	1	14		345789
467	5	2	3	1	1	2	2	25	1	1	1	3			126
468	5	1	3	2	2	2	2	2	2	1	1	3			1269
469	5	2	1	4	4	4	3	3	2	5	2	1	2		12
470	7	1	3	3	2	5	3	2	2	3	3	3			25
471	7	2	2	3	2	1	3	36	1	1	1	3			34
472	6	2	2	3	2	2	3	7	2	2	2	3			19
473	5	2	3	1	1	1	1	7	1						19
474	7	1	1	2	2	2	3	7	2	5	2	3			13479
475	3	2	3	2	2	1	3	5	1	1	4	2		34	1239
476	5	2	3	1	1	1	2	25	1	3	1	3			1267
477	4	1	3	1	2	2	2	3	1	1	1	2		3	129
478	2	1	3	2	2	2	3	3	4	3	1	1	12		3
479	5	2	3	1	1	2		3	1	1	1	3			12389
480	3	2	3	1	1	2	2	7	1	3	1	1	4		4
481	4	2	1	2	4	4	4	4	2	1	2	1	134		12345678
482	3	1	3	1	1	1	1	2	1	3	3	3			12
483	5	2	3	2	2	2	2	3	1						9
484	6	2	3	2	2	2	2	2	2						49
485	3	2	3	1	1	1	3	35	2	3	3	3			129
486	5	2	3	3	3	3	3	7	1	1	1	1	4		123568
487	5	2	3	1	2	2	3	2	2	3	3				1
488	3	2	3	1	1	1	1	2	1	1	1	3			2
489	7	1	2	2	3	3	2	346	1	2	3	1	3		239
490	7	2	1	3	3	4	4	1	1	5	1	1	24		139
491	6	1	1	2	4	3	2	6	2	1	1	2		4	237
492	5	1	2	2	3	3	2	7	1	3	2	1	3		23689
493	6	1	1	3	3	5	2	7	2	1	1	1	1		3
494	5	1	3	1	1	1	1	5	2	5	2	3			589
495	5	2	3	2	2	1	2	356	2	3	2	3			789
496	6	2	2	1	2	2	2	5	1	2	1	1			12345679
497	6	2	1	5	5	5	5	6		1	1	1	23		
498	7	2	1		3	1	2	3456	1	1	1	1	13		1345679

499	4	1	3	2	1	1	2	7	1	1	1	3			45789
500	4	1	3	2	1	1	2	7	1	1	1	3			45789
501	5	2	1	5	3	3	4	4	2	1	1	1	23		68
502	2	1	3	3	2	3	4	3	2	3	3	3			
503	6	1	2	1	1	1	1	1	1	2	1	1	34		12345678
504	5	2	1	2	2	2	2	1	1	2	2	1	13		
505	4	2	1	5	4	3	4	46	1	1	1	1	123		46789
506	4	1	3	1	1	1	1	7	1	5	5	3			9
507	5	2	3	1	1	1	2	57	1						28
508	8	1	1	1	1	2	2	3	1	5	2	3			1249
509	6	2	2	3	3	4	2	246	2	3	3	3			15689
510		2	1	2	1	1	2	25	1	5	2	1	234		139
511	4	2	3	2	1	1	2	2	1	3	3				129
512	4	2	3	2	1	1	2	2	1						9
513	7	2	2	3	3	3	3	3	2	2	1	1	134		39
514	8	1	2	2	3	3	3	3	2	2	2	3			23679
515	4	1	3	1	1	1	1	1	1	2	1	1	123		134789
516	5	2	1	3	5	5	5	346	2	3	3	3			279
517	7	1	1	2	2	2	2	3	2	2	1	1	123		139
518	6	2	2	3	3	3	4	7	1	1	1	1	13		9
519	4	2	3	2	2	1	2	5	1	2	1	1	4		12
520	7	2	1	5	3	2		7	2	2	2	3			2468
521	3	1	2	3	3	3	4	3	2	3	3	3			1
522															
523	6	2	1	1	4	4	3	6	2	1	1	1	2		3469
524	5	2	2	2	5	5	5	34567	2	3	2	3			245689
525	7	1	1	4	4	4	2	7	1	5	3	1	4		125
526	2	2	3	1	1	1	1	5	1	4	2				28
527	6	2	2	2	3	3	2	3	1	2	2	1	13		139
528	4	2	1	1	2	3	2	4	2	2	1	1	2		2789
529	4	1	1	5	2	1	1	34567	2	2	2	3			129
530	5	1	3	2	2	3	2	7	1	1	1	1			
531	6	1	3	2	2	2	2	3	2	3	1	1	4		1478
532	7	1	1	5	5		3	346	2	4	4	2		1	26
533	5	2	1	3	3	3	4	4	1	1	1	1	3		9
534	5	1	1	2	4	4	3	3	2	2	1	1	3		5679
535	4	2	1	3	1	1	2	3	2	4	2	1	1		5689
536	6	2	2	3	3	3	2	46	2	1	1	3			12345689
537	6	2	3	3	3	3	4	34	1			1	1		1269
538	7	1	2	1	4	4	4	456	2	1	1	3			3
539	6	2	1	1	3	3	2	346	1	1	1	3			245689
540	5	1	3	1	1	1	1	3	1	1	1	1	12		1234
541	3	2	3	4	4	3	5	3	2	1	1	2		4	489
542	5	1	1	2	3	3	1	7	1	4	1	1	3		47
543	4	1	1	2	3	4	4	3	2	1	1	1	3		12345678
544	3	2	3	3	2	2	2	3	1	1	1	1	1		12345678

545	4	2	1	3	3	3	3	4	1	1	1	1	13		69
546	2	2	3	1	1	1									
547	7	1	2	1	2	3	1	3	2	2	1	3			156
548	6	2	2	4	4	3	1	34	2	1	1	1	3		24689
549	3	1	1	2	3	2	3	3	1	2	1	1	3		4
550	5	2	2	2	4	4	5	2	1	2	3	2		4	124589
551	7	1	1	4	4	3	3	34		4	3	1	3		68
552	7	1	1	4	4	3	3	34		4	3	1	3		68
553	5	1	2	2	2	2	1	3	1	4	1	2		4	3
554	7	1	1	3	3	3	3	6	1	1	1	1	1		12345678
555	2	2	3	4	3	2	3	346	1	2	4	3			136
556	3	1	2	4	4	3	4	36	2	5	2	1	13		129
557	4	1	3	1	2	3	3	3	1	5	1	3			1257
558	6	1	2	4	4	4	4	3456	2	2	2	3			239
559	5	1	3	3	4	5	4	346	1	5	1	1	1		25689
560	4	1	1	2	4	3	3	3	1	1	1	1	3		348
561	3	1	1	4	3	2	3	346	3	2	2	2			1
562	5	1	1	4	4	4	4	346	2	2	2	1	13		35689
563	5	2	2	2	2	2	2	5	2	3	2	3			12346789
564	5	2	3	3	3	3	2	3	2	5	1				389
565	5	1	1	4	4	4	2	346	2	1	1	1	13		34678
566	5	1	1	2	4	4	4	3456	2	1	2	1	1		3789
567	5	1	1	1	1	2	2	3	1	2	2	2		2	4
568	5	1	1	3	2	3	4	4	1	4	2	1	34		156
569	5	2	1	2	4	4	4	3456	1	4	2	1	134		2349
570	8	2	1	4	4	3	4	356	1	1	1	1	134		124689
571	5	1	1	1	4	3	3	4	3	2	2	1	1		4
572	3	2	1	5	5	4	4	4	1	1	1	1	13		678
573	3	2	1	5	5	4	4	4	1	1	1	1	13		678
574	3	2	3	2	3	1	2	7	1						124
575	5	1	3	4	3	4	3	7	2	5	3	3			3478
576	4	1	2	2	3	3	3	3	1	1	1	1	3		123
577	6	1	1	2	3	3	2	36	2	2	2	3			12458
578	1	2	1	1	1	1	3	3	1	3	4	3			12
579	2	1	1	3	4	4	4	3	1	1	1	1			7
580	4	2	1	1	1	1	1	3	2	4	2	1	4		9
581	5	1	1	1	2	3	2	7	1	3	3	3			57
582	6	1	3	1	3	2	3	57	1	3	2	3			1238
583	5	2	1	1	3	4	5	6	1	1	1	1	12		124689
584	4	1	1	4	4	4	2	1	2	2	2	1	123		2
585	3	2	1	1	1	1	1	3	1	2	4	2		4	1
586	5	2	1	4	5	5	4	6	1	1	1	1	3		8
587	6	1	1	3	3	2	2	6	2	2	1	3			13
588	6	2	1	5	5	5	5	3							
589	2	2	3	1	1	1	1	5	1	5	1	1	4		138
590	7	2	1	3	3	3	5	3	2	2	2	1	3		146

591	3	2	1	3	3	3	2	34	1	1	1	1	13		24689
592	3	1	2	3	3	2	3	36	1	1	1	2		3	23468
593	3	2	1	3	3	3	3	3	2	1	1	1	1		1245689
594	5	1	2	3	2	4	2	3	1	2	1	2		3	1256789
595	4	1	2	2	4	3	4	4	2	2		1	1		1
596	6	1	2	3	3	1	2	7	1	2	1	1	3		9
597	7	1	2	2	2	3	2	6	2	1	1	2		3	12468
598	3	2	3	2	2	1	2	35	1						2
599	4	1	1	1	2	1	1	3	1	2	1	1	1		1
600	7	1	1	4	4	3	4	3456	2	1	1	1	1234		8
601	5	2	1	2	2	2	3	3467	2	2	2	1	135		12345678
602	5	2	1	4	4	4	4	46	2	1	1	1	13		14579
603	3	2	1	2	2	3	1	3	1	1	1	1	25		12345678
604	5	2	3	3	3	1	3	24	1	3	1	1	134		12345678
605	4	2	1	4	4	4	2	46	1	1	1	1	13		12456789
606	4	1	1	4	4	5	2	346	2	2	3	1	134		12345678
607	5	1	3	1	1	1	2	57	1	3	3	3			12
608	6	2	1	4	3	4	1	46	2	2	1	3			4
609	3	1	3	1	2	2	2	35	1	1	1	3			36

MS, medication status; D/FA, difficulty in falling asleep; A/MID, awakening in the middle of the day; A/EM, awakening in the early morning; DD, daytime disfunction; M/V, motivations for viewing the video; C, comprehension; M/BV, motivation to reduce medication before viewing the video; M/AV, motivation to reduce medication after viewing the video; WC, willingness to consult with their doctor about medication reduction; RA, reasons for choosing “agreed”; R/D, reasons for choosing “disagreed”; Imp, impressive video content
